# Managing Ethical Challenges to Mental Health Research in Post‐Conflict Settings

**DOI:** 10.1111/dewb.12076

**Published:** 2015-01-08

**Authors:** Anna Chiumento, Muhammad Naseem Khan, Atif Rahman, Lucy Frith

**Keywords:** research ethics, empirical ethics, mental health, emergencies, post‐conflict, humanitarian

## Abstract

Recently the World Health Organization (WHO) has highlighted the need to strengthen mental health systems following emergencies, including natural and manmade disasters. Mental health services need to be informed by culturally attuned evidence that is developed through research. Therefore, there is an urgent need to establish rigorous ethical research practice to underpin the evidence‐base for mental health services delivered during and following emergencies.

This paper discusses ethical challenges to conducting mental health research in a post‐conflict setting and puts forward possible solutions. Drawing upon a South Asian case study we identify six ethical challenges that were encountered. Each challenge is discussed in relation to wider ethical standards of research practice, and the applicability of existing normative frameworks to a post‐conflict context is critically assessed. Our discussion emphasises the situated nature of responses to ethical challenges encountered during the research.

We then explore recent proposals for managing ethical issues in global health research, identifying their relative strengths and weaknesses. We conclude by calling for documenting and reflecting upon empirical evidence of research practice to stimulate consideration of procedural ethics and ethics in practice. This process aims to promote a moral discourse that can contribute to the development of ethical research practice to underpin mental health research in emergencies.

## Introduction

Emergencies include natural disasters, man‐made disasters, and (protracted) refugee or internally displaced persons (IDP) settings. They create a range of problems at the individual, family and societal levels, including mental health and psychosocial problems.^1^ Given the exceptional nature of emergencies, mental health research is required to build evidence of effective, acceptable and feasible services for contexts where mental health conditions may be aggravated by experiences of disaster and displacement.^2^ Such research, as with all human subjects research, must be ethical and maintain high standards of researcher integrity.^3^ Consequently, the need to confront the ethical challenges inherent to conducting mental health research in emergencies is clear.

Both human rights and research ethics are concerned with normative standards and make claims about how humans *ought* to be treated in certain situations. They emerged from a shared history of rights violations stimulating an international human rights regime,^4^ and guidance relating to medical ethics.^5^ These guidelines codify the normative standards which healthcare research must uphold.

Post‐conflict mental health research is more likely to occur in low‐ and middle‐income countries (LMIC). It is therefore important to acknowledge that ethical and human rights norms, arguably, are premised upon a Western Liberal tradition that prioritise individual rights^6^ and may clash with non‐western conceptions. Through increasing international collaborative – including interdisciplinary – research, standards are homogenised, typically through importing research ethics codes from developed nations to LMIC.^7^ As Emerson et al. highlight: ‘the “is” of those living in the developing world is not the same as the ‘is’ of those living in industrialised nations, and this is morally significant’.^8^ In light of this, this paper questions the uncritical application of Western ethical research standards to community‐based emergency contexts in LMIC, arguing that a more nuanced view of what ethical research ‘looks like’ is needed.

Whilst ethical research standards can be viewed as imported from another setting, they provide a useful starting point for critically considering existing norms of research practice. Drawing upon a case study we discuss the difficulties of importing standards and propose strategies for managing ethical issues in research conducted in a LMIC post‐conflict context. We put forward a case for critical reflexivity when conducting research in emergencies, examining what ‘ethical research’ entails procedurally and in‐practice.^9^ Procedural ethics denotes the processes involved in applying for and securing formal research ethics approval; whereas ethics in practice refers to day‐to‐day ethical issues that are often not addressed or anticipated when applying for ethical approval.^10^ It has been observed that it is in the application of ethical principles that differences in the way they are interpreted and balance are revealed,^11^ further highlighting the tension between procedural and in‐practice ethics.

Considering ethical research through a procedural / in‐practice lens is particularly illuminating in emergency settings where ethics in practice may be complicated by a range of political, ethnic, economic, social and cultural factors, and where specific procedural guidance on ethical research do not exist. An example of this is carrying out and documenting informed consent in cross‐cultural post‐conflict contexts where socio‐cultural norms and a potential climate of fear must be appropriately responded to. This is often presented in procedural documents as ordered and unproblematic, whereas in‐practice this process can be far more nuanced, requiring gatekeeper as well as individual consent and overcoming privacy and confidentiality fears to documenting consent. The potential disjunction between procedural and in‐practice ethics raises questions about the purpose of procedural ethics for aiding study preparation, as well as the implications when there are significant in‐practice deviations from what is outlined in procedural documents. It is this tension that this paper seeks to explore and propose solutions to.

We argue for moving away from a procedural rose‐tinted presentation of the implementation of ethical principles that obscures in‐practice realities, instead encouraging engagement and debate on how ethical challenges inherent to mental health research in emergencies are managed. The approach, referred to in this paper as ‘empirical ethical reflection’ proposes a process to support ethical decision making in which ethical norms are specified from abstract principles to applied contexts, clarifying and converting ethical theories into guides for action.^12^ The empirical ethical reflection approach proposes a framework for active engagement with procedural and in‐practice ethical issues that arise in post‐conflict mental health research that is ongoing from research inception to dissemination (see box 2). It is proposed as a potential way to address the procedural / in‐practice tension that this paper draws out. It is important to note that the empirical ethical reflection approach outlined in this paper is under development and will be refined (and potentially renamed) over time.

This paper presents a mental health research case study conducted in a LMIC post‐conflict setting. The key ethical challenges are identified and discussed in relation to existing normative frameworks, critically assessing their applicability to LMIC post‐conflict settings, and by extension – emergency – research. In the final section of the paper a broad outline of the proposed empirical ethical reflection approach is provided, calling for integration of documenting and reflecting upon empirical evidence of research practice to foreground procedural ethics and ethics in practice.

## Case Study

This case‐study outlines an exploratory mixed‐method mental health research study conducted in a post‐conflict setting in South Asia. It is drawn from reflections of the research lead (MNK), academic supervisor (AR), and local research team. Details have been abstracted to protect on‐going research.

The mental health study targeted perinatal women through a community health centre in one district of a South Asia country. It involved a qualitative assessment, baseline quantitative survey, developing and delivering an intervention, conducting an exploratory randomised control trial and follow‐up qualitative interviews. Research was conducted over two years by a local researcher and study team trained and supervised by senior mental health researchers from the South Asian country. The study received full in‐country and UK University ethical approvals.

Military operations officially ended prior to study commencement, but a strong military presence remained with checkpoints and patrols. The community contained active non‐state insurgents, with isolated incidents perpetuating instability. This post‐conflict context produced a number of ethical challenges to research conduct.

BOX 1: The case study examines the management of six ethical challenges:

**Table:** 

Challenges	Ethical issues	How ethical issues were managed
*Who conducts the research?*	Affects access to participants, acceptability and accountability of the research team, participant paranoia and mistrust, and carries implications for research capacity‐building.	Access, researcher accountability and local capacity building addressed by research led and conducted by a local research team comprised of community residents. Research supervision conducted by nationals of the South Asia country experienced in mental health research in complex community settings.
*Who funds the research?*	Disclosure of research funders in research information is accepted ethical research practice. It is important to be aware of local perceptions of funders and the impact this may have upon research participation.	Funded by a national Higher Education body equipped with local knowledge, able to judge study appropriateness for the target population.
*Ethical review*	Ethical review is an accepted procedure to verify the ethical grounding of proposed research.	Local in‐country ethical approval secured prior to obtaining UK University approval, deferring to local assessment. Protocol developed with full participation of the local research team acting as cultural brokers.
*Voluntary informed consent:*	Informed consent is a guiding norm of ethical research practice: human subjects *should* be informed about the nature and implications of research, their rights in the research process, and that participation is voluntary.	Voluntary written informed consent of female participants was required. Cultural norms require prior gatekeeper consent from families. Cultural adaptations to the consent process sought to ensure consent was informed, voluntary, adhered to ethical standards, and was compatible with local cultural norms.
*Community mistrust:*	Mental health research requires unbiased data to guide design, delivery and evaluation of interventions.	Research was shared and agreed with local community representatives to counter misinformation about the study. Community re‐engagement was conducted to address rumours and mistrust.
*Risk to the research team:*	Participant and researcher safety is a guiding principle of research: Do no harm.	‘Do no harm’ was applied to research participants and the research team. Risk to the research team was managed by ‘pauses’ to research activities and community re‐engagement.

## Maintaining Ethical Standards

In this section we discuss the six everyday ethical challenges encountered when conducting research in a post‐conflict setting that were raised in the case‐study, examining existing ethical research standards. We then outline proposed strategies for managing the ethical issues raised by conducting research in emergencies. We conclude by considering the benefit of empirically studying researchers’ experiences for contributing to ethical mental health research in emergencies.

#### Who Conducts the Research?

In social research gauging an appropriate distance between researcher and participant – neither too familiar nor too distant^13^ – is important for methodological rigour. Often the ‘appropriate’ distance is enmeshed with adherence to cultural norms, and is therefore affected by who conducts the research. This is important in mental health research where effective interventions require attention to the cultural context in which participants are embedded.^14^ A local research team who can advocate for culturally centred interventions,^15^ and research processes that respond to cultural context can aid acceptability of services and research.

Who conducts research carries implications for research capacity building, defined as the ability to conduct, manage, disseminate, and apply research in policy and practice.^16^ Gaps in LMIC mental health research capacity have been identified at every level: individual, organisational and national.^17^ The lack of sufficiently trained and experienced local researchers carries ethical implications when research is conducted by those unfamiliar with local context or without sufficient expertise to maintain ethical research standards.^18^ Consequently, the benefits of building local capacity should not be underestimated,^19^ including better integrating LMIC perspectives into research agendas and practice in global health.^20^ Capacity building requires long‐term investment and commitment – including recognising research as a viable career.

Developing local capacity and partnerships also provide routes for initial contact with communities that can increase the acceptability of those conducting research. The way communities are approached has been highlighted as critical to ‘ethical entry’^21^ appropriate to local cultural norms. Partnerships with organisations embedded within local communities also provides routes for researcher accountability to the community.^22^ When negotiating access to communities, particularly in conflict or post‐conflict situations, awareness of power relations and who is identified to represent a community are pertinent.^23^ The potential for researchers to be perceived as supporting one side or another, or privileging certain accounts requires careful attention^24^ and can be exacerbated by researchers working outside of local community systems. Researchers must remain mindful of who they are provided access to, and issues that might arise from only engaging those with the power to speak out, further disempowering those without a voice.^25^ Responding to these considerations is contextual, favouring a situated approach to how ethical entry is managed.

Local research teams are well placed to manage researcher safety. Craig et al.^26^ identify race, gender and culture as potentially impacting upon researcher safety in violent contexts. They advocate addressing safety by matching these and other important characteristics of the research team with the community. The issue of researcher safety is discussed in more detail below.

Therefore, for a range of ethical reasons it is maintained that ethical research conduct requires the incorporation of local researchers within the project team.

In the case study researcher matching and ethical entry were ensured through a local research team, critically incorporating local females including two mothers. These female researchers shared important characteristics with participants and were widely respected within the community. Gender matching researchers and study participants increased the acceptability of the research, strengthening mutual trust and rapport. Appropriate distance between participant and researcher was assured by adherence to local cultural norms such as dress codes including observing purdah (the practice of females wearing a veil and being segregated from men who are not family) and speaking the local language. Researchers were not personally known to participants, but came from the same region, deemed important for building trust and ensuring confidentiality.^27^ Ethical entry was achieved through partnership with a local organisation and respecting cultural norms by discussing the study with community elders and health workers prior to its commencement. Negotiating access to female research participants required attention to gender power imbalances as well as cultural norms relating to decision‐making authority (discussed under ‘informed consent’ below). Ongoing activities in the research site continue to draw upon the skills and expertise of the trained research team, contributing to local capacity building and embedding local partnerships for future research.

#### Who Funds the Research?

The manner in which research is funded in emergencies carries at least two important ethical considerations: first, in post‐conflict situations is the funding source, or country associated with the source, seen to be party to the conflict? This could put researchers and participants at risk of harm. Second, do funding structures enable research that leads to tangible improvements in participants lives in the short and long term?

In post‐conflict settings who fund the research can lead to positive or negative views of the study depending upon local populations’ perceptions of outside agencies. This is particularly pertinent when bodies perceived as party to the conflict fund research, and can impact upon researcher safety.

The question of disclosing research funding raises competing ethical duties. On the one hand there is a duty to develop mental health interventions by conducting research which requires funding, and there is an established ethical obligation to disclose funding sources to participants. On the other hand following this ethical obligation could potentially put researchers at risk. Therefore two ethical principles come into conflict – the disclosure of funding sources to participants, and the duty to ensure researcher safety. To address this we recommend conducting an assessment to consider the ways funding agencies may be viewed by the local community, and how this may impact upon researcher safety. Assessment findings should be shared with funders to negotiate an approach to funding disclosure, considering adjustments to the obligation for full disclosure. This approach is not without its problems. For example, could failure to fully disclose funding sources be viewed as deception, presenting a risk should it become known that researchers were not open with participants? Resolving these tensions requires an approach that accounts for local factors, and not a ‘one size fits all’ ethical requirement.^28^

A further issue is the ethical obligations of funders. Schopper et al. highlight ‘reasonable availability’ of an intervention post‐research, defined as a commitment to deliver services for a minimum of two years, or that it remains available through other means.^29^ Similarly, the *Council for the International Organisation of Medical Sciences (CIOMS)* identify as ‘morally praiseworthy’^30^ the sponsor funding services beyond the duration of research, with this commitment outlined in research protocols; guidance that is reflected elsewhere.^31^

The issue of research funding should be included within ethical risk / benefit analysis. In the field of mental health research benefits typically include influencing the design and delivery of services. This is the aim of WHOs ‘Building Back Better’ which advocates strengthening health systems in the immediate aftermath of emergencies for long‐term benefits.^32^ However, policy process can be lengthy meaning participants may not see research impact. This is particularly the case where intervention delivery is tied to short‐term funding rather than a commitment to embed services into routine care. Delays in research impact or short‐term services can create poor perceptions of research participation.

The Hastings Centre consider the duty of ‘fair benefits’ from research participation as laid out in CIOMS.^33^ They argue that ‘fair benefits’ is poorly operationalised, with lack of clarity over who is to benefit (research participants, the wider community, a whole country?) and who is responsible for funding this benefit (research funders, governments, international organisations?). They support the community deciding the value of fair benefits of research participation, and what these should entail. Therefore, ethical research is not tied to continued access to services, but could include benefits such as capacity building of local service providers or researchers, contributing to health infrastructure, or financial reward. This raises a duty for researchers and funders to engage with communities to determine how benefits can be ethically distributed, delivering immediate and long term benefits of value to the community.

In the case study the issue of who funds the research was minimised as funding came from a National Higher Education body and a recognised local NGO. These funding sources increased local acceptability of the research and were fully disclosed to study participants. Future research benefits included an intervention provided by embedded health workers which continued to be delivered beyond the research, and capacity building of both healthcare providers and researchers. These benefits were discussed informally with healthcare providers prior to research, exploring how to embed research into existing services. Capacity building was viewed as of particular benefit and involved training healthcare staff at two centres in the mental healthcare needs of perinatal women.

However, in light of community mistrust it is possible that some participants considered the local funding sources as a route for government authorities to extract information regarding involvement in insurgency activity. Therefore, it is maintained that an assessment of the socio‐political context be conducted, including in internal conflicts where community allegiances may lead to local funders being viewed with suspicion.

#### Ethical Review

Research ethics review is a procedural cornerstone of international guidelines on human subjects research.^34^ However, lacking or dysfunctional review boards in many LMICs contribute to inadequate ethical research standards.^35^ Challenges ranging from review boards’ legal status, workloads, and differences in expertise and procedures contribute to disparities in the review process.^36^

Developing research ethics committee members capacity is frequently highlighted as a way of ensuring reliable interpretation of international ethical guidelines for socio‐economic and cultural conditions.^37^ This concurs with calls for in‐country review to judge ‘ethical acceptability of the research in accordance with the customs and traditions of the community’, involving lay persons to review research against community cultural and moral values.^38^

Advocating formal ethical review is premised on the view that when conducted well feedback can be instrumental to ensuring research maintains ethical standards. A subsidiary aim is to stimulate a conversation between researchers and reviewers, seeking consensus on how to manage potential ethical issues. In the ethical review process it is important to recognise informal community‐level procedures for reviewing research that operate alongside formal review, ensuring that the latter does not usurp the former: conversations with ethical review boards should not replace conversations with communities involved in research. An iterative process between ethical review boards and communities to identify, define and negotiate the way ethical challenges will be resolved is recommended. This approach problematizes the priority of formal ethical review, with the strongest process being one that balances formal review with community‐led processes. An iterative approach acknowledges that most ethical issues arising in research implicate a number of principles which requires a process of judging the relative weight to be accorded each principle.^39^

In complex contexts it has been suggested that those conducting ethical review are often in a ‘double‐bind’: they recognise the risks and potential for exploitation, but have little practical guidance to offer on the management of ethical issues.^40^ This suggestion concurs with recent research identifying the paucity of guidance from ethical review committees on a study conducted with IPDs.^41^ Involving the community to collaboratively design research, including developing responses to potential ethical issues, as well as having community review of research, offer potential mechanisms to address this ‘double‐bind’.

Challenges to iteratively developing specified ethical standards for international mental health research are recognised, not least of all the time required. Another difficulty is the complexity of ethical review at multiple levels – community, in‐country, international and increasingly funder review. However, these processes aid development of ethical standards for specific studies that adhere to both local and international norms, and support researcher preparedness by thinking through ethical issues prior to data collection. Multiple levels of review can also stimulate ethical review committees cross‐learning: educating international committees on country context, culture and moral values; in‐country committees can see how international committees work; and both local and international committees can learn from community responses to potential ethical issues. Therefore, due to potential long‐term benefits for specific research studies and a broader moral conversation we argue for using the opportunity of engaging with multiple levels of review and iterative development of ethical standards to build examples of best practice for managing potential ethical issues in a range of contexts.

Whilst this proposal could be charged with being idealistic, it is countered that researchers routinely engage with procedural demands which frequently entail multiple levels of ethical review, for example in the country where the study is to be conducted and in the sponsor country. This demonstrates that with sufficient planning and researcher commitment multi‐level review is possible. A further challenge is presented when seeking to follow the process of multi‐level review in settings where no local review structures exist. In this case we recommend a peer‐review assessment of the protocol either by local academics, practitioners or community members to ensure research is critiqued from a local perspective.

In this case study formal ethical review was obtained both in‐country and at a UK University. Community perspectives were represented by the locally‐based researchers who took an active role in developing the study protocol and acted as cultural brokers, identifying potential ethical challenges and suggesting routes to manage these – such as the informed consent process discussed below. Additionally, sharing proposed research with community health workers and elders provided informal community‐level review. Contrary to the above discussion on the opportunities for cross‐learning through ethical review processes this was not experienced in this study. Approval at all levels was provided without comment on the potential ethical issues that may arise. Therefore, management of in‐practice ethical issues relied heavily upon informal local review and comment, researcher integrity, and knowledge of the study setting rather than formal ethical review processes. It is not known if this missed opportunity is a result of those conducting review feeling they were in a ‘double‐bind’ or due to poor capacity and review procedures.

#### Voluntary Informed Consent

Informed consent is a guiding norm of ethical research practice: human subjects *should* be informed about the nature and implications of research, their rights in the research process, and that participation is voluntary.^42^ Informed consent arose from legal standards of physician duty towards research participants, and contemporary moral theory which conceptualises the patient as subject.^43^ It is premised upon the moral notion that rational people will choose to do what is good for them.^44^ Homan^45^ identifies four elements to voluntary informed consent:

1.  All pertinent aspects of what is and might occur are disclosed;
2.  The participant should be able to comprehend this information;
3.  The participant is competent to make a rational judgement;
4.  Agreement to participate should be voluntary, free from coercion and undue influence.

Research guidelines recognise informed consent in LMIC raises additional cultural considerations, including the issue of gatekeepers^46^ and differing conceptualisations of ethics and rights.^47^ Attending to power relations is also identified,^48^ with one study seeking to mitigate power hierarchies’ related to religious, community and political leaders, as well as the status accorded to medical professionals and researchers which can create undue inducement to participate.^49^

In this case study gatekeeper consent was conducted, respecting local cultural norms. This entailed obtaining prior consent from household males and elders to seek consent from the female participant. This can be viewed as taking consent from multiple levels or ‘spheres’,^50^ including whole communities, community leaders or elders, families, and individuals as appropriate in the setting. This process presents ethical risks as it is possible that gatekeepers may not allow an individual to participate. In this circumstance the participant is unable to exercise their right to make an informed choice to participate. This presents an ethically charged dilemma for researchers balancing adherence to cultural context with ethical and human rights norms.

Chambliss suggests informed consent ‘represents at best a polite fiction’,^51^ a view pertinent to emergency contexts.^52^ In the case study before research assistants sought informed consent the research was introduced by a community health worker who provided a short explanation of the study and asked permission for a research assistant to meet with the individual. Through training, research assistants’ self‐awareness of the impact disparities in education and status could have upon making an informed choice to participate were raised, and the participants right to refuse to participate without penalty was reinforced. Therefore, each step in the informed consent process sought to protect participant rights whilst remaining compatible with local cultural norms.

Additional safeguards were also considered including taking repeated consent, an approach adopted through repeat verbal consent. This compromise aimed to minimise raising anxieties in relation to the research purpose, and formed one aspect of addressing community mistrust through consistent articulation of the research process, discussed below. A challenge to repeat consent is that it could promote higher attrition rates, something particularly relevant to randomised control trials.

Relating to procedural ethics, in the case study, due to high rates of illiteracy all research information was explained verbally in the local dialect with a thumb print accepted in lieu of a signature, following standard practice in the context. It is important to note that in different settings a thumb print can itself carry negative connotations. Other alternatives for recording consent with illiterate populations are to record verbal consent or have researchers witness and verify consent on behalf of participants. However, both strategies present ethical challenges. In some contexts, including the case study, recording is not acceptable to the local community or presents risks to confidentiality. Equally, to have researchers verify consent on participants’ behalf can be considered insufficient protection against coercion. Consequently, decisions relating to how to record consent must be carefully examined with local researchers who can act as ‘cultural brokers’ to ensure acceptability of consent processes.

In the case study despite providing information in the local language and attempting to overcome illiteracy through verbal explanations of the research, rumours of threats to the research team called into question how far confidentiality, anonymity, and protection of participant rights was understood. Difficulties translating concepts such as anonymity and confidentiality into the local dialect raise questions as to the meaning participants ascribed to them. Perceptions that interviews are collecting information to pass onto intelligence authorities are more likely to occur where the population feel threatened, such as conflict or post‐conflict settings. This indicates that whilst consent processes can be culturally adapted, they may be unreliable when undertaken with an illiterate population who feel threatened.

Accordingly, the case study consent process in some cases failed to meet Horman's element 2: comprehension of research information.^53^ This raises a critical ethical dilemma: how to ensure information is fully comprehended at the time of obtaining consent? Moreover, what are the implications for consent should it transpire that information was not fully comprehended? These strike at the heart of the principle of informed consent, and have been discussed elsewhere.^54^ Strategies for managing this in LMIC have been proposed including: placing emphasis upon the process of information exchange over formal recording of consent;^55^ providing information in lay language appropriate to local literacy,^56^ where required including images or video to aid communication;^57^ and conducting an oral examination to gauge sufficient knowledge to make an informed decision about participation.^58^ The first two strategies offer routes to enhance the quality of information exchange and are deemed appropriate in emergencies. An examination to verify comprehension is deemed inappropriate given the potential for disempowering potential participants. However, the principle of asking participants to reflect back in their own words their understanding of research has been recommended^59^ and is considered a less formal approach to verifying study comprehension to make an informed decision about participation. Furthermore, relating to information, concerns about the way information is constructed and presented have been raised.^60^ This is important in emergencies where clear and unbiased presentation of information is critical to avoiding exploitation.

As this brief discussion emphasises, the practice of ensuring voluntary informed consent is frequently complex, requiring researchers to judge the quality of consent. It may only be once research is underway that it emerges to what extent the information provided during consent was understood by participants. Therefore, ethical standards may require acknowledgement of situations where it may not be possible to obtain fully informed consent due to contextual realities such as a climate of fear. In such circumstances a more nuanced view of consent may be appropriate with alternative guarantees of ethical research practice. In this regard the biomedical field could learn from the work of social scientists and anthropologists.^61^ The possibility of negotiated consent involving collaboration with the community and flexibility as to what consent ‘looks like’ by research ethics review boards (i.e. not dependent upon signing a form), is believed to offer potential for more culturally appropriate and robust consent processes. This more nuanced view is felt to be suitable for emergencies.

#### Community Mistrust

Managing paranoia or mistrust over the way information collected during research is to be used requires careful handling in communities exposed to conflict. Craig et al. identify that it can be necessary to equip researchers with tools to respond to strong feelings or angry reactions to research by participants and the local community.^62^ This is particularly important in mental health research where stigma and discrimination are common, reinforcing the importance of a trained research team.^63^

Promoting trust requires active communication and mutual understanding between researchers and the community. At a minimum communities should be consulted during the research planning stage, on an ad‐hoc basis whilst the research is conducted, and provided with research findings in an appropriate format and timely manner.^64^ In addition, care should be taken to distinguish between routine care and participating in research in order to avoid therapeutic misconception.^65^ These measures are essential to reducing community misperceptions about research.

In the case study community mistrust was an important ethical concern. When going to homes in the community researchers were confronted with families fearful for their safety. On rare occasions researcher safety was threatened when rumours of threats against the research team spread as a result of questions relating to exposure to violence. With families active in the insurgency these questions were interpreted as collecting information to pass onto intelligence authorities, carrying implications for participant recruitment and increasing risk to the research team perceived as acting at the behest of the government.

These issues were managed by (a) integrating local community members into the research team, and (b) suspending research activities whilst mistrust was addressed by the study lead, a precaution designed to ensure researcher safety. The locally based study lead and researchers ensured an ‘ear to the ground’, seeking to anticipate community mistrust and respond accordingly. In the post‐conflict study setting the integration of local researchers increased the credibility of claims that research was not collecting information for intelligence authorities.

During the suspension of research the study lead conducted repeated research information events with those making threats. This involved detailing the broad study topic, what participation entailed, how information would be used including protection of participant anonymity and that personal information would remain confidential to the research team. Opportunities for questions and discussion were provided. In addition, as identified above, researchers conducted repeat verbal consent, reinforcing key principles of voluntary participation and protection of participant rights. The presentation and re‐presentation of information sought to counter community rumours and mistrust, re‐engaging participants and the wider community in research. This strategy proved effective in this context. Therefore, the response to this ethical issue was locally specific, developed by embedded researchers in accordance with local cultural norms.

#### Risk to the Research Team

Research investigating sensitive topics needs to assess the potential risks research poses to both participants and researchers.^66^ This is equally important when researchers are working in sensitive contexts such as emergencies. Examples of safety risks include threats to physical safety; risk of psychological distress; potential for accusations of improper behaviour; and increased exposure to everyday risks such as infectious illnesses or accidents.^67^

Risk assessments are an integral element of developing a research protocol, including budgeting and planning to manage potential risks to participants and researchers.^68^ Managing risks to the research team is essential to ethical research which is dependent upon researcher competency to practice,^69^ including addressing stress and fear. Guidelines to support development of protocols to manage researcher safety have been suggested. These include steps to assess the situation, identifying and responding to threats, and developing preventative strategies and follow‐up procedures,^70^ including addressing potential psychological harm.^71^ Psychological support referral pathways for research teams are essential when conducting research with populations exposed to violence or trauma, or where upsetting or difficult disclosure may arise. In addition, in emergencies processes for consulting local security experts^72^ as well as those coordinating the emergency response are also essential.

Recognising the potential emotional and psychological impacts of discussing sensitive topics is important to preventing researcher burnout.^73^ Mental health and trauma‐related research may lead to researchers experiencing vicarious trauma: traumatisation through the act of bearing witness to the suffering of interviewees.^74^ Equally, failure to ask these questions results in an incomplete picture of mental health status and may miss cases of abuse or degradation, hence can be viewed as unethical not to ask.

Adequate attention to personal and psychological safety of researchers requires specialist training of research teams that emphasises strategies for researcher self‐care, supervision and support.^75^ Such training addresses the concerns of Dunn that ‘the novice researcher is usually taught that the research process is orderly and straightforward’.^76^ They also offer an opportunity to bridge the gap between procedural ethics and ethics in practice, engaging with potential real‐world difficulties that may arise in research conduct. Training therefore promotes a more nuanced approach to the way in which specific ethical challenges will be managed.

In the case study support was provided though daily meetings between the study lead and the research team. In these meetings the research team detailed the day's field activities and reported any events that had happened. These collective meetings provided opportunities for peer support and raising concerns. They also ensured the study lead was aware of field challenges and could monitor researchers’ wellbeing. In addition to these daily meetings the study lead was available via mobile phone for immediate contact in the cases of crisis.

One crisis arose during the case study where military raids of homes occurred whilst interviews were being conducted. This resulted in research team members becoming fearful for their safety, compounded by high profile insurgent activity targeting women and health workers. These threats to safety were managed through pausing the research for one week so as not to coincide with activities being targeted by insurgents. Given the similarity of the study to activities being targeted – a health campaign conducting house‐to‐house calls – this was felt to be appropriate, with research resuming only after the other activities had ceased.

This highlights the ethical duty to be flexible in the research schedule, suspending research to not coincide with activities of a similar nature being targeted in order to protect both researchers and participants. This carries ethical implications when projects are externally funded as hostile activity may prevent a study being concluded, including withdrawing when a mental health intervention is ongoing which may leave participants at risk, and the ethical implications of perceived wasted financial resources. Despite the potential risks it is important that mental health research with groups or in settings perceived high risk is conducted^77^ which requires robust risk management. This necessitates an approach to ethical research practice that responds to risks inherent to different contexts, employing culturally appropriate strategies to address and mitigate risk to ensure that research adheres to the principle ‘do no harm’. Sharing strategies for responding to risk in different contexts will promote a moral conversation to identify best‐practice approaches to minimising risks.

## Research Ethics in Emergencies: Arguing for Specified Normative Frameworks

Specific approaches and epistemological positions for responding to ethical issues encountered in the conduct of global mental health research have been proposed by various authors. These will be considered and suggestions made for incorporating greater empirical ethical reflection to support ethical research conduct in post‐conflict and emergency settings. We argue for the development of a nuanced ethical discourse on research practice in emergencies that responds to specific issues that arise in certain types of studies or in particular contexts.

Siriwardhana et al.^78^ propose a post‐study ethical audit to evaluate researcher integrity and decision making that could have compromised the ethical grounding of research. What this audit would entail, who would be involved and the degree to which this would deliver genuine critical reflection upon ethical issues requires further elaboration. However, this proposal could form a useful addition to the research cycle, promoting reflection upon management of ethical issues and evaluating procedural ethics against in‐practice realities. This process offers the opportunity to stimulate wider learning by researchers, ethical review committees, and potentially communities.

An addition to the post‐study audit could be pre‐study reflection to promote preparedness. This would differ from existing approaches to research planning which focus upon procedural ethics, instead encouraging active engagement and reflection upon in‐practice ethical challenges that may be encountered. This process should include the community to identify ethical considerations relating to context such as adherence to local cultural or religious norms. Open engagement with potential ethical issues is anticipated to increase research team preparation for in‐practice challenges that may be obscured by a purely procedural approach to research planning.

The Ethical, Cultural and Social Program for Global Health proposes addressing issues ‘up‐stream’ in the research process through Consultation Services in Research Ethics.^79^ These committees of experts in research ethics at academic bioethics centres provide advice and guidance about ethical issues that arise in the design and conduct of research. In this process the role of local experts is privileged, and the overarching aim is to build upon specific cases to propose solutions to cross‐cutting issues. Such services can stimulate moral conversation and address issues of capacity in research ethics review. However, they remain abstracted from research‐in‐practice, and their success is largely predicated upon their ability to overcome the ‘double‐bind’ where risks and the potential for exploitation are recognised, but practice suggestions as to how these can be overcome are unavailable to the reviewer.^80^ Therefore, integration of the community into this process to promote knowledge about the way ethical principles are balanced in different settings is recommended, moving away from expert academic driven strategies and towards an ethos of co‐learning.

Iltis et al.^81^ identify considerations relevant to risk communication and management to support the ethical conduct of mental health research. They focus upon both procedural management and communication of risks in ethical approval applications, as well as supporting active engagement with in‐practice ethics through adequate training and preparation of researchers. They call for further research into risk communication and management, learning from critiques of research information, ensuring research information and consent processes accurately portray study risks and benefits and do not unduly bias views of the research. They cite the ethical imperative of justice as placing the duty upon the research community to ‘design ethically and scientifically sound research that does not ignore populations or kinds of research merely because of the difficulties involved’,^82^ a call relevant to mental health research in emergencies. Whilst a useful proposal that engages with the procedural / in‐practice disjunction, we argued that the focus upon risk of harm should be balanced against potential benefit, and should be extended to include risks to researchers.

Addressing the ethical issue of who and what are studied and why has recently been considered through a social justice lens. Feminist approaches have been suggested as a framework for health research that attends to social justice, emphasising multiple and complex structures of inequality and power.^83^ These acknowledge the impact of keeping those affected by multiple forms of oppression on the margins of society, health, and research.

Rogers and Kelly highlight that researchers’ involvement in the subjective worlds of participants can reveal knowledge related to health disparities and systemic inequalities. This approach offers a useful critique of existing normative frameworks that can silence moral discourses emerging from local communities. It attends to research‐in‐practice, foregrounding power, discrimination and social justice; as well as procedural ethical review, where Western norms and review processes frequently take priority over LMIC^84^ or informal community processes. Viewed through a social justice lens the extent to which normative frameworks of ethical research reify structures of inequality and power is emphasised. Adopting this approach to ethical research offers one response, recognising the non‐absolute nature of ethical decision making and that norms are subject to contextual application.^85^

Each of these approaches emphasise ethical issues raised by conducting global health research. These issues are necessarily magnified emergencies in which problems of social justice and inequity are exacerbated, structures of dependency are prevalent, and existing family, community and societal support are disrupted.^86^ In such settings the imperative to ensure research is conducted ethically is paramount. It has been argued that the ethical conduct of research does not equate to importing ethical norms and standards of practice that may be inappropriate to culture and context. Ethical research practice is nuanced, premised largely upon researcher training, experience and above all integrity. To recognise this and to engage with the ethical issues raised by conducting mental health research in complex settings, moving away from rose‐tinted protocols and towards addressing real world in‐practice challenges, is a much needed bold step the research community must be prepared to take.

We propose that this process requires empirical ethical reflection. This entails active engagement with ethical issues procedurally and in‐practice that is ongoing throughout the research process – from inception to dissemination. This should include pre‐research planning involving local communities alongside researchers that seeks to unmask potential ethical issues that may arise to enhance protocol writing, researcher training and study preparedness. In research conduct and dissemination efforts should be made to capture and document researchers’ experiences of applying ethics in practice, revealing potential deviations from what was outlined in procedural documents. Findings from this process should be used to consider potential implications for the validity and reliability of research findings. Learning from these processes should be disseminated, recognising that reliable research is a product of ethically sound research planning and conduct and that researcher's should report on all study limitations, including those that relate to ethics. This broad approach offers sufficient flexibility to integrate and address the shortcomings of the above proposals.

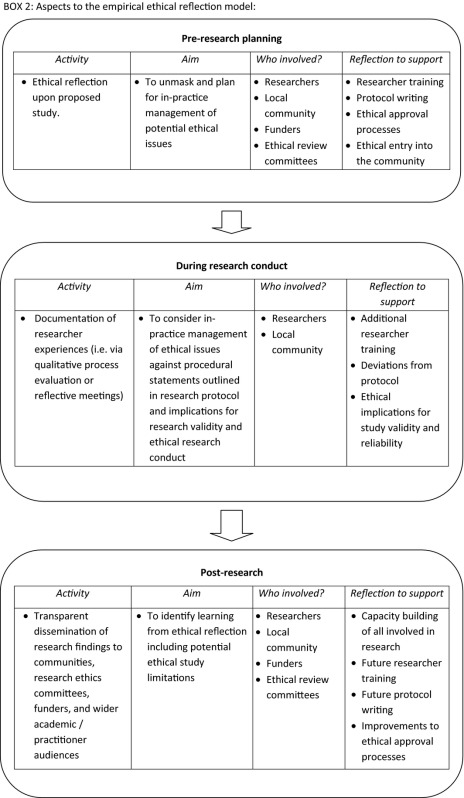

We illustrate this proposal with the hypothetical example of disclosure of research funding. This hypothethical research is conducted in a post‐conflict setting where military presence remains, including indiscriminate arrests, causing community mistrust and paranoia. Funding comes from a government / private funder collaboration known as GHR, which is managed and distributed by an internationally recognised charity, REGA. The research is conducted by a consortium involving a local NGO and international Universities and Health Organisations.

Funders require their sponsorship be disclosed to participants. Direct reference to GHR is therefore inserted into procedural documents (protocol and participant information sheet). This is discussed at a meeting prior to submission for ethical approval, and following advice from the local NGO that the funder is viewed with suspicion due to perceived involvement of the Government in the recent conflict, this is amended, stating ‘funding for this study comes via REGA’. This approach is discussed with local community representatives who feedback that the study would be more readily accepted through reference to a local body, such as the NGO implementing the research. Therefore, to strengthen local accountability further reference is made to the study being led by a recognised local NGO. This balanced approach is approved by ethical reviewers and research funders, who recognise the value of this compromise in the local setting.

This approach is discussed at field researcher training, and emphasis placed upon following the text in the information sheet. The question is raised as to whether the researchers should disclose the funder is GHR if participants ask the question. A detailed discussion sought to balance the need for transparency with the potential risk to researchers and the study if full disclosure were to lead to hostility towards the research. The compromise agreed to respond to the question by identifying the funder as ‘a western collaborative involving government and private funders’. This approach was recorded in notes about the training which formed one element of the process evaluation. It was applied by field researchers and found to be effective. However, in field researcher supervision it was discovered that those who asked for additional detail on study funding were more likely to decline participation than those who did not, documented as part of the process evaluation. No further observations or issues relating to funder disclosure were raised during research conduct.

At the end of the research a reflective meeting was held, involving all research investigators and field researchers. At this meeting the approach taken to funding disclosure was critically reflected upon, revisiting procedural documents and in‐practice findings from the process evaluation which led to identifying competing ethical duties of accountability, transparency, and researcher safety. The higher levels of non‐participation amongst those more aware of the funding source rose whether the research had in misled participants. It was concluded the rationale for not fully disclosing the funding source was an overriding ethical duty to uphold the principle of ‘do no harm’. Furthermore, the information provided to participants was deemed accurate; it was just not as detailed as it could have been. It was also observed that the paranoia and mistrust of GHR was based upon misleading media coverage, and therefore difficult for researchers to counter. Finally, all agreed that the ethical duty of conducting needed research was implicated, with providing much needed intervention services and long‐term community benefit through capacity building of local health workers and researchers justifying the compromise taken in this instance. This procedural / in‐practice learning was documented along with other empirical ethical reflections in a short report to funders and ethical review committees who had approved the study; and was reported in more detail through a conference paper reproduced as a reflective article published in a peer‐reviewed international journal.

As this hypothetical example illustrates, much of the in‐practice ethical decisions are aspects of day‐to‐day research management. However, when identified as ethical issues and reflected upon from an ethical perspective the rationale and limitations behind ethical decisions is rendered explicit. The benefits of empirical ethical reflection therefore become clear: active reflection upon decisions relating to research conduct that carry ethical implications increases transparency and builds evidence of the way ethical principles are flexibly applied in specific settings.

Whilst this process requires additional resources and potentially time for the conduct of studies, it is argued that these are acceptable to achieve ethically robust research practice. A key limitation to these approaches when conducted as internal self‐monitoring exercises is the willingness of researchers to actively reflect and identify potential limitations to ethical research practice. However, it must be observed that principles of transparency and critical reflection are central to all research conduct, therefore it is felt that this limitation can be overcome.

## Conclusion

Some key ethical considerations when conducting research in emergencies have been highlighted through discussion of a relevant mental health case study. These are in no way unique to the context in which the case‐study was conducted, although the potential risks were higher than could be expected in peacetime. We suggest one response to ensuring ethical research practice is for researchers to engage in empirical ethical reflection entailing engagement with research practice on both descriptive and normative planes. Documenting and reflecting upon experience aims to promote the emergence of a moral discourse around the way ethical principles can be implemented and promoted in research conducted across cultural contexts.

As demonstrated in the case study, reflection upon empirical evidence of research practice can stimulate important ethical considerations. Through examination of research practice empirical ethics approaches aid critical consideration of background assumptions of moral principles, such as informed consent.^87^ Given the range of ethical concerns raised in this paper, it is suggested that interrogation of research practice through an empirical ethics lens could support better understanding and management of the ethical implications integral to conducting mental health research in post‐conflict and by extension emergency settings. To render explicit the practice of attaining ethical research in a given context will enhance learning. This recognises that ‘the effort to ensure that research is conducted ethically [necessitates] a thoughtful process of balancing ethical considerations [which] can be as important as any particular judgement’.^88^ Therefore, we call for moving away from rigid implementation of ethical principles and towards appreciating the fluid processes of ethical research in practice. This is not to reject existing normative frameworks, but to call for a considered approach to their application that recognises that ethical research conduct is not a product of adherence to a set of rules, but of a mutually respectful encounter.

Importantly, most frequently missing from research reports are the experiences of those on the ground, ‘too little attention is given to documenting the process of carrying out research’.^89^ We call for increased attention to documenting this process, building empirical evidence that critically considers the ethical difficulties in undertaking mental health research in complex contexts and with complex populations. In this way, global initiatives can contribute to development of an approach to applied ethics that responds appropriately to the specific issues raised in practice and promotes ethical standards to underpin research practice in emergencies.

## Funding

This work was supported by funding from a Higher Education Commission and local NGO to NK. The corresponding author (AC) is supported by the Economic and Social Research Council [ES/J500094/1].
